# Vitamin D receptor activation in microglia suppresses NOX2‐mediated oxidative damage via PAT1 in vitro and in vivo

**DOI:** 10.1002/ctm2.1187

**Published:** 2023-01-23

**Authors:** Changshui Wang, Yahao Gao, Beibei Chen, Pei Jiang

**Affiliations:** ^1^ Department of Neurosurgery Affiliated Hospital of Jining Medical University Jining Medical University Jining China; ^2^ Clinical Medical School Jining Medical University Jining China; ^3^ ADFA School of Science University of New South Wales Canberra Australia; ^4^ Institute of Translational Pharmacy Jining Medical Research Academy Jining China; ^5^ Translational Pharmaceutical Laboratory Jining First People's Hospital Shandong First Medical University Jining China

Dear Editor,

Vitamin D (VD) is established to have both neurotrophic and neuroprotective effects.^1^ Understanding the regulation of calcitriol (active form of VD)^2^ proteome may provide new insights into the neuroprotective mechanisms of calcitriol and could also provide fundamental new knowledge regarding the underlying molecular signalling. Proteomics analysis (Figure 1A) identified 7 up‐regulated and 28 down‐regulated proteins following calcitriol treatment in BV2 microglia (Log_2_FC ≥ .58 or Log_2_FC ≤ −.59, *p* < .05). Gene ontology functional annotation of differentially regulated proteins showed that they are involved in molecular functions, including cellular anatomical entity, biological regulation and binding (Figure 1B). In addition, KEGG pathway enrichment analysis demonstrated that proteins regulated in response to calcitriol administration were involved in immune responses and metabolism (Figure 1C). One of the most strongly down‐regulated proteins was PAT1 (Log_2_FC = −1.218) (Figure 1D). The results of PAT1 protein expression were consistent with the proteomics findings (Figure 1E). In addition, VD receptor (VDR) expression was significantly increased following calcitriol treatment. Sequence and binding site analyses revealed that VDR was a candidate transcription factor regulator of the PAT1 promoter region (Figure 1F) and verified by luciferase assays and chromatin immunoprecipitation (Figure 1G,H). We found that calcitriol treatment markedly reduced luciferase activity, and that VDR can directly regulate PAT1 transcription.

**FIGURE 1:**
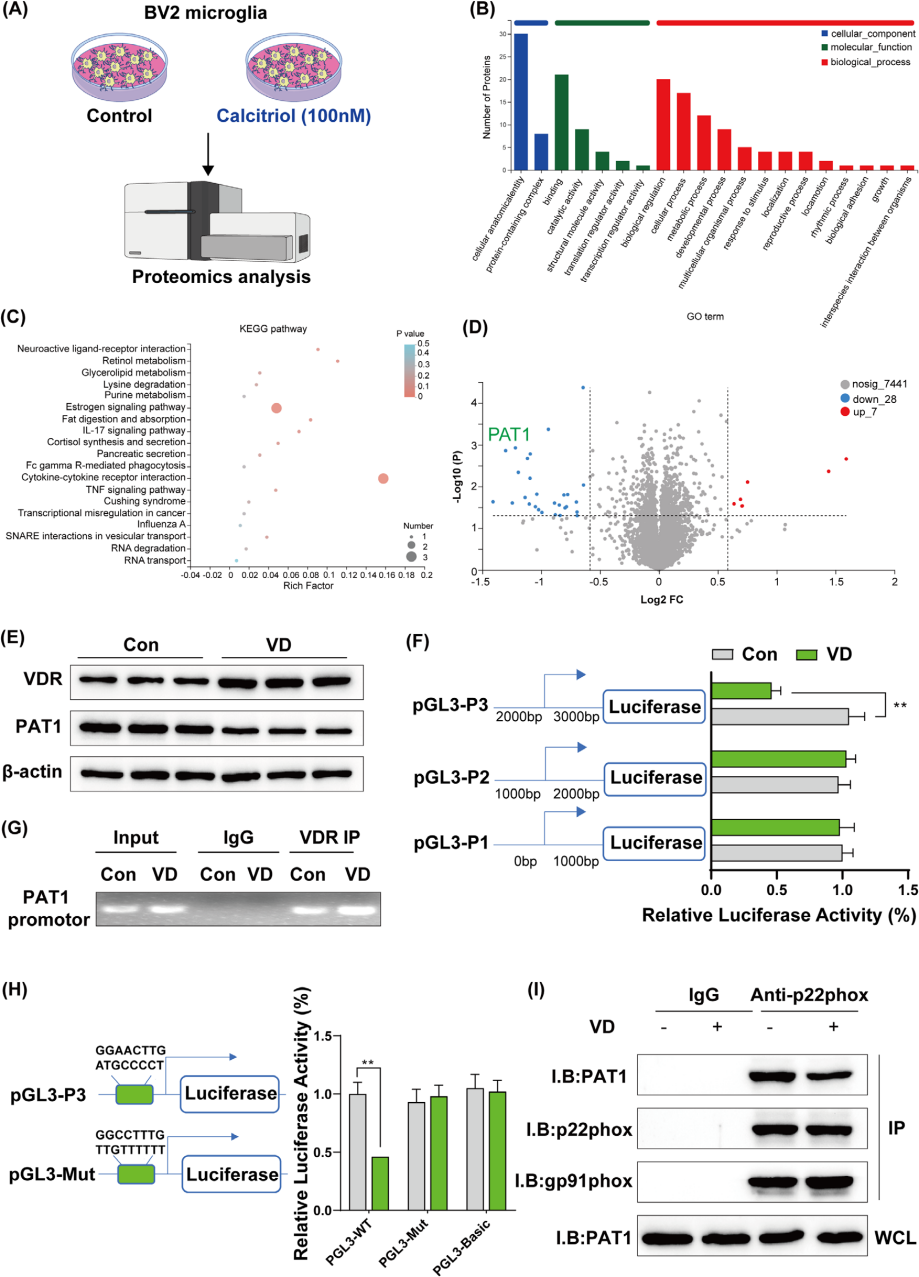
(A) Schematic diagram of the in vitro experiment. (B) Gene ontology analysis of differential expression protein. (C) KEGG pathway enrichment analysis of differential expression protein. (D) Volcano plot of differential expression protein. (E) Representative Western blot of vitamin D receptor (VDR) and PAT1 protein expression levels. (F) Luciferase activity was analysed in different segments of promoters of the PAT1 with or without VD treatment. (G) Chromatin immunoprecipitation analysis of the association of VDR with the PAT1 gene promoters. (H) Schematic diagram representing the binding sites of gene promoters and mutants as well as luciferase activity. (I) Proteins were immunoprecipitated from BV2 cells lysates with anti‐p22phox Ab or control IgG and analysed by Western blot with anti‐PAT1, anti‐p22phox and anti‐gp91phox. The data are expressed as mean ± SD (*n* = 3). **p < .01

A recent study identified PAT1 as a potential partner of NOX2 in human neutrophils and monocytes, with an important role in the regulation of NADPH oxidase activation.^3^ To determine whether PAT1 also interacts with NOX2 in microglia,^4^ we performed immunoprecipitation analysis. Interestingly, PAT1 was detected in p22phox (the transmembrane subunits of NOX2) immunoprecipitates, whereas no PAT1 was detected in the control IgG group (Figure 1I). Additionally, Western blot analysis showed that calcitriol administration caused a significant decrease of PAT1/p22phox binding.

We also observed co‐localization of PAT1 and p22phox using confocal microscopy. Our data show that PAT1 was translocated to the plasma membrane and co‐localized with p22phox when stimulated with lipopolysaccharide (LPS),^5^ whereas calcitriol administration significantly reduced this phenomenon (Figure 2A), suggesting that VDR activation inhibits PAT1 transcription and subsequently inhibits the interaction of PAT1 with NOX2. Next, we prepared extracts of cell components to evaluate the expression levels of related proteins in various subcellular locations. In response to LPS exposure, PAT1 levels at the membrane increased significantly. In parallel, membrane levels of the NADPH oxidase p47phox subunit were also increased following LPS stimulation, whereas PAT1 gene interference had a similar inhibitory effect on LPS‐induced membrane translocation to calcitriol treatment (Figure 2B and Figure S1A–D). These results strongly support interaction between PAT1 and NOX2 and inhibition of this effect by calcitriol treatment.

**FIGURE 2:**
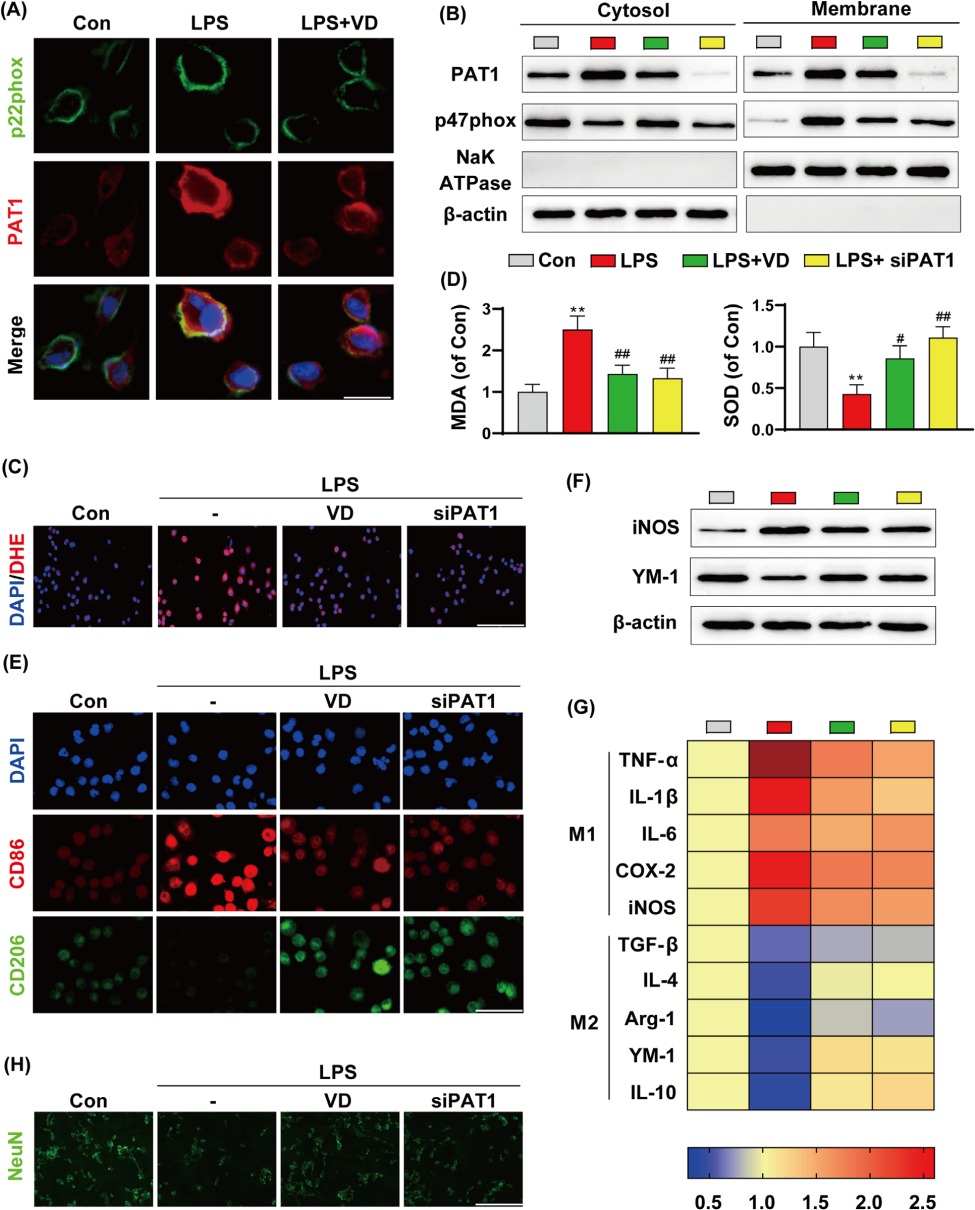
(A) Representative confocal microscopy images of p22phox and PAT1. Scale bar: 20 μm. (B) Proteins were extracted from the cytosol and membrane and analysed by Western blot with anti‐PAT1, anti‐p47phox. (C) Representative images of immunofluorescence assays of dihydroethidium (DHE). Scale bar: 200 μm. (D) Content of lipid peroxidation product, malondialdehyde (MDA) and antioxidant enzymes, superoxide dismutase (SOD) activity. (E) Representative images of immunocytochemistry assays of CD86 and CD206. Scale bar: 100 μm. (F) Representative Western blot of pro‐inflammatory iNOS and immunoregulatory YM‐1 protein expression levels. (G) Heat map of pro‐inflammatory and immunoregulatory mRNA expression levels. (H) Representative images of immunocytochemistry of NeuN staining. Scale bar: 200 μm. The data are expressed as mean ± SD (*n* = 3). **p < .01 compared to control group. #*p* < .05, ##*p* < .01 compared to lipopolysaccharide (LPS) group

p47phox translocation from the cytosol to the membrane is a key step in the activation of NADPH oxidase, which produces reactive oxygen species.^6^ Intracellular ROS levels increased significantly after LPS treatment, whereas calcitriol pretreatment or PAT1 knockdown, markedly reduced ROS levels (Figure 2C). In addition, LPS treatment significantly increased the levels of malondialdehyde (MDA) and decreased the activity of superoxide dismutase (SOD) compared with controls, whereas these effects were reversed by calcitriol or PAT1 knockdown (Figure 2D). Subsequently, we examined the polarization state of microglia. Immunofluorescence and immunoblotting analysis showed that LPS induced microglia to promote M1 polarization and inhibit M2 polarization. Interestingly, calcitriol pretreatment or PAT1 deficiency induced the transformation of microglia from M1 to M2 phenotype (Figure 2E,F and Figure S1E,F). Immune responses in the brain are closely related to microglial polarization,^7^ and calcitriol addition or PAT1 silencing effectively decreased the mRNA expression levels of pro‐inflammatory factors and increased those of anti‐inflammatory factors (Figure 2G). Further, to determine whether calcitriol has a protective effect on neurons, we co‐cultured neurons with microglia. Staining with the neuronal marker, NeuN, revealed that LPS treatment resulted in substantial neuronal loss and apoptosis, relative to the control group and NeuN‐positive cells (Figure 2H) and TUNEL‐positive cells (Figure S1H,G) were restored by calcitriol pretreatment or PAT1 silencing.

Next, we investigated whether calcitriol can regulate PAT1 expression in C57BL/6J mice (Figure 3A). Interestingly, calcitriol treatment (.5 μg/kg/day by i.p. for 14 days) reduced PAT1 and NOX2 expression by LPS (1 mg/kg i.p. every other day for 14 days). Consistent with our in vitro findings, LPS stimulation decreased levels of p47phox in the cytosol but increased them in the membrane fraction. Further, calcitriol or PAT1 deficiency attenuated LPS‐induced PAT1 and p47phox membrane translocation (Figure 3B and Figure S2A–F). Accordingly, calcitriol treatment or PAT1 deficiency significantly decreased NADPH oxidase activity induced by LPS. Moreover, calcitriol also decreased MDA levels and increased SOD and catalase activities (Figure 3C). These results suggested that VDR activation by calcitriol treatment suppresses the activity of NOX2 and alleviates oxidative stress via PAT1 inhibition.

**FIGURE 3:**
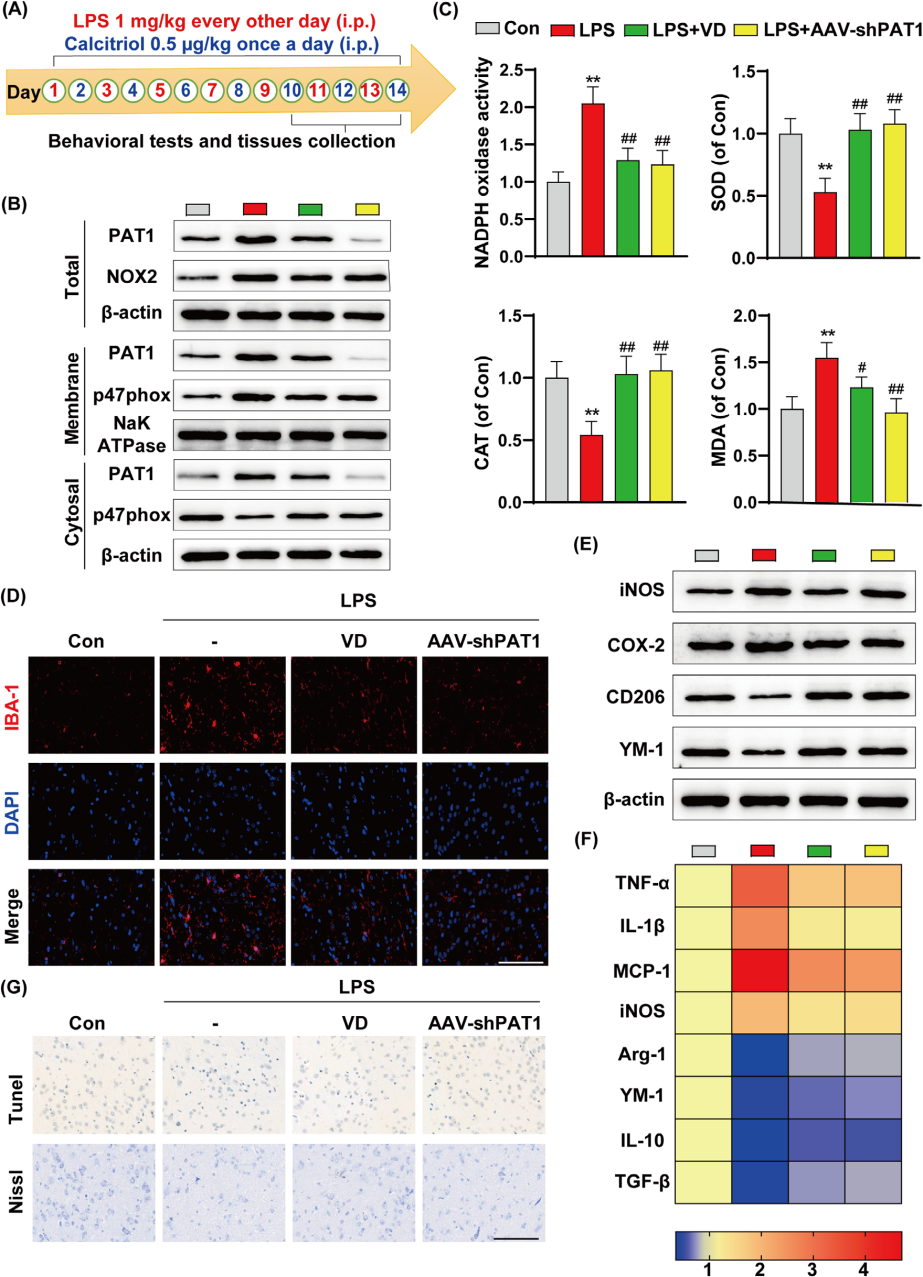
(A) Timeline of the in vivo experiments. (B) Proteins were extracted from the cytosol and membrane and analysed by Western blot with anti‐PAT1, anti‐p47phox. (C) The activity of NADPH oxidase and the antioxidant enzymes, superoxide dismutase (SOD), catalase (CAT) as well as content of lipid peroxidation product, malondialdehyde (MDA). (D) Representative images of immunofluorescence assays of Iba‐1. Scale bar: 100 μm. (E) Representative Western blot of pro‐inflammatory and immunoregulatory proteins expression levels. (F) Heat map of pro‐inflammatory and immunoregulatory mRNA expression levels. (G) Representative images of Tunel and Nissl staining. Scale bar: 200 μm. The data are expressed as mean ± SD (*n* = 3). **p < .01 compared to control group. #*p* < .05, ##*p* < .01 compared to lipopolysaccharide (LPS) group

We then explored the neuroprotective effects of calcitriol in neuroimmune modulation.^8^ Intraperitoneal injection of LPS along with calcitriol significantly decreased the abundance of Iba‐1‐positive cells in the cortex of mice relative to treatment with LPS alone (Figure 3D). A general decrease in the expression of pro‐inflammatory markers and an obvious increase in expression of anti‐inflammatory markers were observed with calcitriol treatment (Figure 3E,F and Figure S2G–J). Furthermore, LPS induced an increase in TUNEL‐reactive cells stained brown and a significant decrease in Nissl bodies in the cortex. Consistently, calcitriol treatment or PAT1 interference markedly relieved these effects and improved neuronal survival (Figure 3G).

Neuronal damage is often accompanied by severe cognitive and behavioural impairment.^9^ Therefore, we evaluated the effect of calcitriol on the behaviour and cognitive functions of LPS‐treated mice using behavioural tests. In terms of cognitive function, calcitriol treatment or PAT1 interference ameliorated LPS‐induced increases in successfully reaching a hidden platform and was associated with more frequent platform crossing and longer time in the target quadrant after platform removal (Figure 4A,B and Figure S3A,B). Regarding mouse neurobehavioral abnormalities,^10^ LPS exposure resulted in decreased central exploration time in the open field test (Figure 4C and Figure S3C), a decrease in entry into the open arm of an elevated maze (Figure 4D and Figure S3D,E), decreased sugar water intake in the sucrose preference test (Figure 4E) and increased immobile time during forced swimming (Figure 4F). Interestingly, calcitriol treatment or PAT1 interference resulted in different degrees of behavioural abnormalities.

**FIGURE 4:**
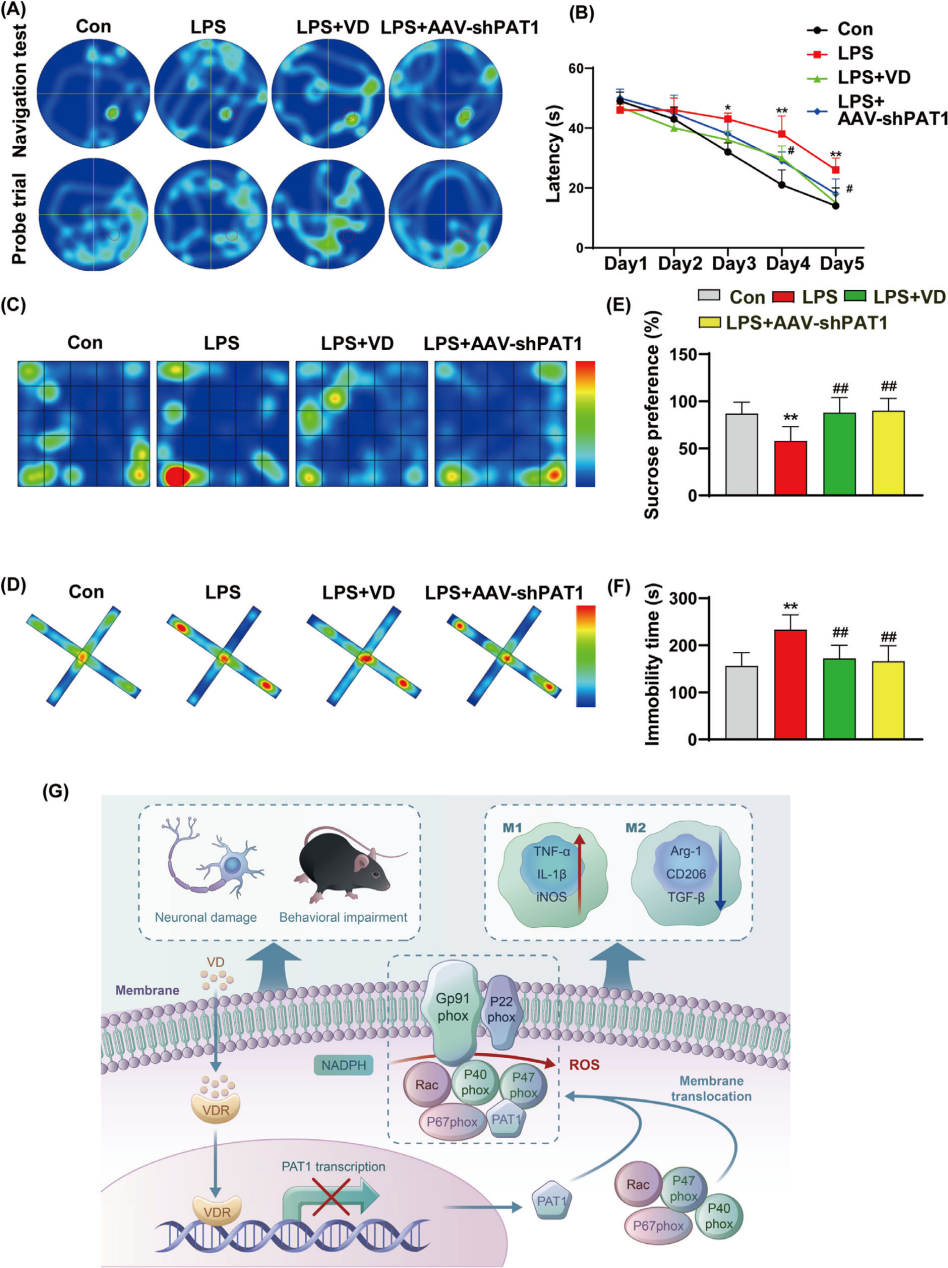
(A) Representative trace depicting the paths of the mice in orientation navigation test and in spatial probe test, as well as (B) the escape latency. (C) Representative trace depicting the paths of the mice in open‐field test. (D) Representative trace depicting the paths of the mice in elevated plus maze test. (E) The sucrose preference in the sucrose preference test. (F) The immobility time in force swimming test. (G) Schematic diagram of molecular mechanism of vitamin D receptor (VDR) activated by VD‐regulating PAT1 against oxidative damage. The data are expressed as mean ± SD (*n* = 6). **p < .01 compared to control group. ##*p* < .01 compared to lipopolysaccharide (LPS) group

Overall, our findings showed a novel neuro‐activity of VD, which activates VDR and further mediates the inhibition of PAT1 transcription upon binding to the PAT1 promoter region, leading to a decrease in PAT1 expression and subsequently an inhibition of NOX2 activation (Figure 4G). Therefore, these results may shed fresh light into the antioxidative and anti‐inflammatory mechanisms of VD, providing the evidence for its neuroprotective actions in the inflammation‐related brain dysfunctions.

## CONFLICTS OF INTEREST

The authors declare no conflicts of interest.

## Supporting information

Supporting Information

Supporting Information

## Data Availability

The datasets used and analysed during the present study are available from the corresponding author on reasonable request.
