# Supplementary material for: Vitamin D receptor activation in microglia suppresses NOX2‐mediated oxidative damage via PAT1 in vitro and in vivo
Source: Clin Transl Med. 2023 Jan 23;13(1):e1187. doi: 10.1002/ctm2.1187 (PMC9869429; doi:10.1002/ctm2.1187)
Supplement: Supplementary file 1 — Supporting Information [file CTM2-13-e1187-s002.docx]

**Figure S1**


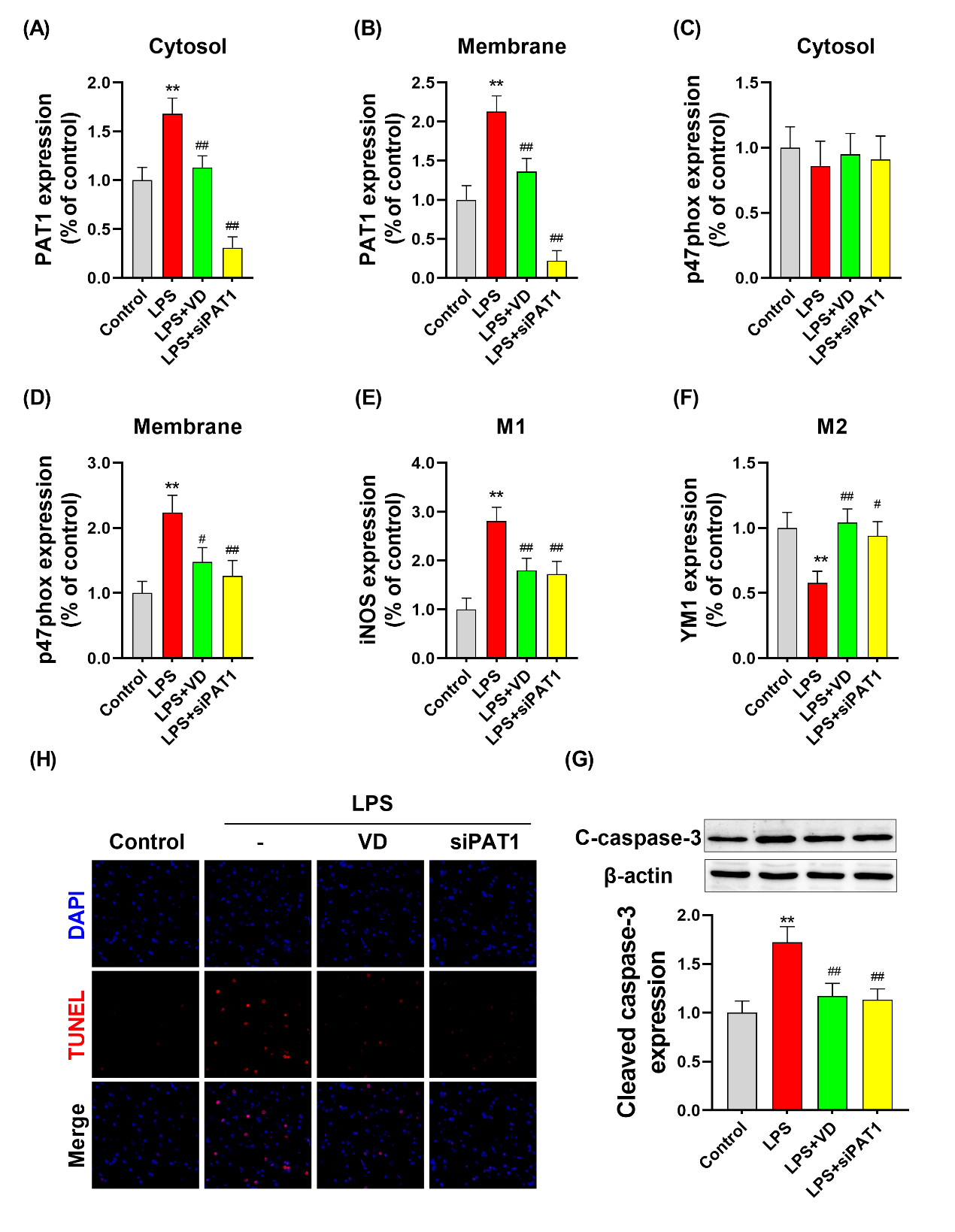
 **Figure S1**

Statistical graphs of protein expression of PAT1 (cytosol A and membrane B), p47phox (cytosol C and membrane D), iNOS (E) and YM1 (F). Representative images of immunofluorescence assays of TUNEL (H). Scale bar: 100 μm. Representative western blots and statistical graphs of apoptosis related protein cleaved-caspase-3 (G). * p < 0.05, ** p < 0.01 compared to control group. # p < 0.05, ##p < 0.01 compared to LPS group.

**Figure S2**
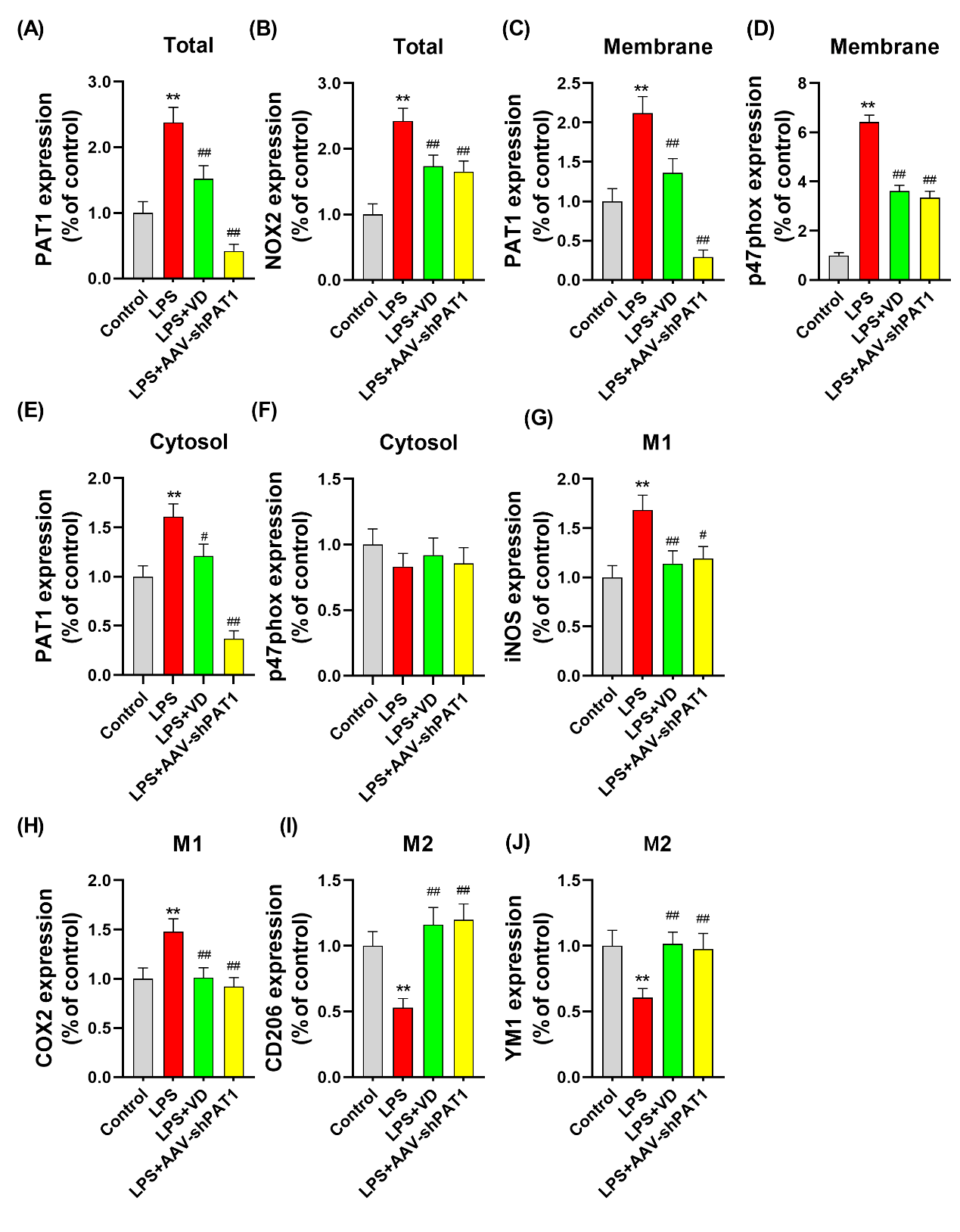


**Figure S2**

Statistical graphs of protein expression of PAT1 (Total A, membrane C and cytosol E), NOX2(B), p47phox (membrane D and cytosol F), iNOS (G), COX2(H), CD206 (I) and YM1 (J). * p < 0.05, ** p < 0.01 compared to control group. # p < 0.05, ##p < 0.01 compared to LPS group.

**Figure S3**


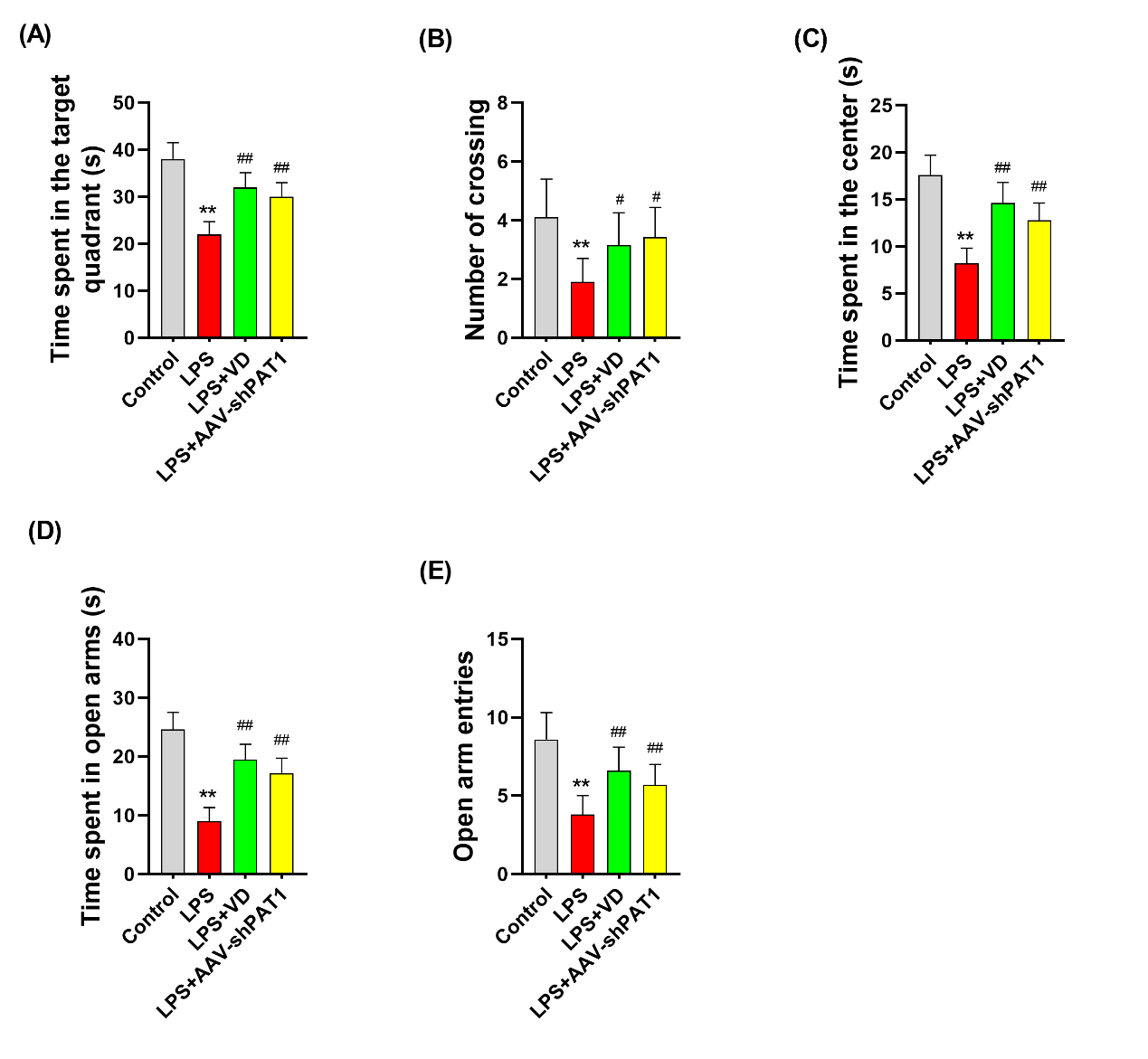


**Figure S3**

Time spent in quadrant in MWM test. (B) Counts of crossings over location where the platform had been placed in MWM test. (C) Time spent in the central area in OFT test. (D) time spent in open arms and number of open arm entries (E). The data are expressed as mean ± SD (n = 6). ** p < 0.01, compared to control group. # p < 0.05, ##p < 0.01 compared to LPS group.
