# Supplementary material for: Vitamin D receptor activation in microglia suppresses NOX2‐mediated oxidative damage via PAT1 in vitro and in vivo
Source: Clin Transl Med. 2023 Jan 23;13(1):e1187. doi: 10.1002/ctm2.1187 (PMC9869429; doi:10.1002/ctm2.1187)
Supplement: Supplementary file 2 — Supporting Information [file CTM2-13-e1187-s001.docx]

**Material and Method**

**Animals and drug administration**

Eight-week-old male C57BL/6J mice were purchased from the Jinan PengYue Experimental Animal Breeding Co. Ltd (Jinan, China). The mice were housed under standard conditions with 12-hours of light/dark cycling and free access to food and water at room temperature (23 ± 2°C). A total of 32 mice were randomly divided into following four groups (n=8): (1) control group (vehicle), (2) LPS group, (3) LPS+VD group, (4) LPS+AAV-shPAT1. Mice received LPS via intraperitoneal injection at a dose of 1 mg/kg every 2 days for a total of seven injections; calcitriol was intraperitoneally injected at 0.5µg/kg for 14 days; for LPS+AAV-shPAT1 group, two weeks after AAV-shPAT1 adenoviral injection, the mice were intraperitoneal injection of LPS (1 mg/kg) every 2 days for 14days. Morris water maze (MWM) training with platform from day 10 to day 13 and the test without platform on day 14. After the behavioral tests, mice were anesthetized with an intraperitoneal injection of 1% sodium pentobarbital (50 mg/kg) and the brain tissues were rapidly removed on the ice surface. All animal use procedures were carried out in accordance with the Guide for the Care and Use of Laboratory Animals and were approved by the Ethics Committee of Jining First People’s Hospital.

**Adeno-associated viral vector (AAV) preparation and injection**

The AAV vector was constructed in Shandong ViGene Biosciences. To knockdown PAT1 expression, the selected shRNA sequence and scrambled control were cloned into the pAAV vector under control of U6-promoter. For stereotaxic injection, mice were anesthetized with isoflurane and fixed to a stereotaxic apparatus. Then, 1 µl of AAV-shPAT1 was slowly injected into the frontal cortex of the fixed mice. The injection was carried out using a 10-μl Hamilton injector fitted with a 30-guage beveled needle at a rate of 0.25 μl/min. Upon completing the injection, the needle was retained in position for 5 min and then gently withdrawn to prevent virus reflux.

**Proteomic analysis**

BV2 cells treated with or without calcitriol were used for proteomic analysis. After extraction, protein was performed quality inspection, enzymolysis, TMT labelling using TMT reagent (ThermoFisher) added with acetonitrile and hydroxylamine, followed by LC-MS/MS analysis. Then, according to the identification of mass spectrometry, all proteins and protein sequences were compared with major databases (Uniprot, NR, GO, KEGG, String) and subcellular localization related databases, and the annotation information of proteins in each database was obtained. Fold Change (FC) and p-value were selected as the reference criteria for the screening of differential proteins between groups. Set FC≥1.5 or FC≤0.66, p < 0.05 as the filtering parameter for differential proteins.

**Primary cortical neuron culture**

Primary cortical neurons were cultured from fetal C57BL/6J mouse brains. Dissection of cortices was done aseptically and minced on ice with scissors. The shredded tissue was digested in 0.25% trypsin (Gibco, Shanghai, China) and 200 U/ml DNase (Sigma-Aldrich) for 30 min at 37 °C. After filtering through a 400-mesh sieve, the suspensions were centrifuged for 5 minutes at 1,000 rpm. The cells were cultured on poly-D-lysine-coated plates for 7 days. Primary neurons were cultured with Neurobasal-A medium supplemented with 2% B27 (Gibco), streptomycin/penicillin (50U/mL streptomycin and 50U/mL penicillin), and 1% glutamate. The medium was refreshed every 2 days.

**BV2 cell culture and treatment**

BV2 murine microglial cells were grown in Dulbecco’s modified Eagle’s medium (DMEM) supplemented with 10% fetal bovine serum (FBS), 1% penicillin/streptomycin at 37°C incubator with humidified atmosphere of 5% CO2. One day before the experiment, BV2 cells were seeded in 6-well and 12-well plates at a density of 1× 10 ^6^/well and 5× 10 ^4^/well respectively. Following one day incubation, BV2 cells were exposed 500 ng/ml LPS for 24 h and with or without 100nM calcitriol treatment. To explore the effect of activated microglia on neuron, the conditioned medium from the BV2 microglia in each group was collected and applied to primary cortical neuron. BV2 cells were transfected with si-PAT1 using Lipofectamine 3000 (Invitrogen) according to the manufacturer’s instructions.

**Chromatin immunoprecipitation (ChIP) assay**

Chromatin immunoprecipitation assay was carried out following the manufacturer's instructions from the ChIP assay kit (Beyotime). Briefly, the proteins were crosslinked to the DNA by adding 1% formaldehyde to the cell medium and incubating at 37°C for 10 minutes, followed by sonication to shear the genomic DNA, and a ChIP-grade antibody against VDR (Abcam, ab109234) was used for immunoprecipitation. The ChIP product was quantified using PCR with specific primers for the PAT1 gene promoters (Table 1). PCR products were electrophoresed in 2% agarose gel and visualized under BioRad Gel Documentation system.

**Co-immunoprecipitation (****Co-IP) and Immunoblotting**

Lysis of cultured cells using the Immunoprecipitation Kit with Protein A+G Magnetic Beads (Beyotime) according to the manufacturer's instructions, followed by Co-IP and analyzed by western blot. Preparation of total protein by lysis of cultured cells or cortex tissue using RIPA buffer (Solarbio). For membrane and cytosolic protein analysis, proteins are extracted using a membrane protein extraction kit and a cytoplasmic protein extraction kit (Solarbio) respectively, according to the instructions provided by the manufacturer. After quantification with BCA kit, proteins were separated by 10% SDS-PAGE gel electrophoresis followed by transfer of the protein to polyvinylidene difluoride membranes by wet transfer cells. The members were blocked for one hour (5% non-fat dry milk) and washed for 30 min (Tris-buffered saline Tween-20, TBST) before overnight incubation of primary antibodies at 4 ℃. Subsequently, three washed with TBST, covered the HPR-conjugated secondary antibody (Cell Signaling Technology, 7074S, 7076P2, 1:2,000) for one hour at room temperature. Protein expression were visualized by the ECL kit (Biosharp, Beijing, China) and the intensity of the blots was quantified by Image J. All antibodies and dilution ratios used are as follows: VDR (Santa Cruz, sc-13133, 1:300), PAT1 (Invitrogen, PA5-106601, 1:1000), p22phox (Santa Cruz, sc-271968, 1:1000), gp91phox (Santa Cruz, sc-130543, 1:1000), p47phox (Santa Cruz, sc-17844, 1:200), Sodium Potassium ATPase (Servicebio, GB11400, 1:500), iNOS (Abcam, ab178945, 1:1000), YM-1 (Abcam, ab192029, 1:2000), Nox2 (Santa Cruz, sc-130549,1:800), CD206 (Proteintech, 18704-1-AP, 1:1000), COX2 (Proteintech, 66351-1-Ig, 1:1000), Caspase-3 (Servicebio, GB11532, 1:1000), β-actin (Proteintech, 66009-1-Ig; 1:4000).

**Real-time quantitative PCR analysis**

Total RNA was extracted from cultured cells or cortex using Trizol reagent (Tiangen, Beijing, China) following the manufacturer’s instructions. Next, RNA was reverse transcribed into cDNA using the FastKing gDNA Dispelling RT SuperMix (TIANGEN). The RT-qPCR was performed on a CFX96 (Bio-Rad, USA) using SuperReal PreMix Plus (TIANGEN). All operations were according to the protocol provided in the kit and performed in triplicates. The 2 ^−ΔΔCq^ method was used to evaluated the relative expression, and β-actin was used to normalize the mRNA levels. Sequences of the primers are provided in Table 1.

**Table 1** Primer sequences used for the qPCR and ChIP analysis

| **Gene** | **Sense Primer (5’-3’)** | **Antisense Primer (5’-3’)** |
| --- | --- | --- |
| TNF-α | GGTGCCTATGTCTCAGCCTC | GCCATAGAACTGATGAGAGG |
| IL-1β | GTGTCTTTCCCGTGGACCTT | TCATCTCGGAGCCTGTAGTG |
| IL-6 | TACCACTTCACAAGTCGGAG | CTGCAAGTGCATCATCGTTG |
| COX-2 | ATGACTGCCCAACTCCCATG | TCTCAGGGATGTGAGGAGGG |
| iNOS | GAGACAGGGAAGTCTGAAGC | CCAGCAGTAGTTGCTCCTCT |
| MCP-1 | ACAAGAGGATCACCAGCAGC | GCATCACAGTCCGAGTCACA |
| IL-10 | CGGGAAGACAATAACTGCAC | CGGTTAGCAGTATGTTGTCCA |
| IL-4 | ATCATCGGCATTTTGAACGAG | ACCTTGGAAGCCCTACAGAC |
| Arg-1 | AGTTGGAAGCATCTCTGGCC | ATCACCTTGCCAATCCCCAG |
| YM-1 | TACTCACTTCCACAGGAGCA | CTCCAGTGTAGCCATCCTTA |
| TGF-β | CCGCAACAACGCCATCTATG | AGCCCTGTATTCCGTCTCCT |
| β-actin | CATTGCTGACAGGATGCAGA | TGCTGGAAGGTGGACAGTGA |
| PAT1 | GTGCCATTTTGTCAGCTGCA | GCACAAGCCCTGCATTTGAA |

**Luciferase reporter assay**

To identify the promoter of PAT1 and VDR binding, the promoter of PAT1 was ligated to the luciferase reporter plasmid. Cells were transfected with reporter plasmid carrying PAT1 promoter and firefly luciferase gene or a PAT1 mutant promoter and firefly luciferase gene using Lipofectamine 2000 transfection reagent (Invitrogen), according to the manufacturer's protocol. After 24 h of calcitriol treatment luciferase activity was measured using a luciferase assay kit (Beyotime, China)

**Oxidative parameters detection**

Dihydroethidium (DHE, Beyotime) for detection of reactive oxygen species (ROS) levels. Cultured cells were incubated with DHE at 37 °C for 30 min and can be observed under an inverted fluorescence microscope after three washed. Superoxide dismutase (SOD), catalase (CAT), NADPH oxidase activity and malondialdehyde (MDA) contents were detected using assay kits (Nanjing Jiancheng Bioengineering Institute, China), respectively, according to the manufacturer’s instructions.

**Histopathological staining**

Immediately after isolation in ice, the brain was fixed overnight in 10% paraformaldehyde and then embedded in paraffin wax. For Nissl staining, prepare 5-μm-thick paraffin sections, after dewaxing the sections were soaked in cresyl violet staining solution (0.1%) for 10 min, rinsed in running water and differentiated in 95% ethyl ethanol. For TUNEL, to detect apoptosis, 5-μm-thick paraffin sections were dewaxed and stained using the TUNEL kit (Beyotime), according to the instructions provided by the manufacturer.

**Immunofluorescence** **assay and confocal microscopy**

Cells cultured on coverslips were fixed with 4% paraformaldehyde for 20 min. Permeabilization in 0.3% (v/v) Triton X-100 in PBS at room temperature for 20 min before blocked with 3% Bovine Serum Albumin (BSA) in PBS for 60 min. For mouse brain tissue, 6-μm-thick paraffin sections were dewaxed in xylene and rehydrated in ethanol series. Antigen retrieval was carried out by boiling the sections in the microwave in a citric acid buffer, next, blocked in 5% goat serum for 60 min at room temperature. The cells or sections, after incubation with the primary antibodies: PAT1 (Invitrogen, PA5-106601, 1:200), p22phox (Santa Cruz, sc-271968, 1:100), CD86 (Invitrogen, 13-0862-82, 1:1000), CD206 (Proteintech, 18704-1-AP, 1:500), NeuN (Abcam, ab177487, 1:100), IBA-1 (Abcam, ab178847, 1:200), washed three times in PBS, followed by incubation with the secondary antibodies, Alexa Fluor 594 or Alexa Fluor 488 IgG conjugate at room temperature for 60 min. DAPI staining solution (Beyotime) was used to stain cell nuclei. Immunofluorescence images were obtained by inverted fluorescence microscopy or confocal microscope

**Morris water maze test**

This assay was conducted in a plastic pool (diameter of 120 cm and height of 50 cm) with opaque water (24 ± 1 °C). The maze was separated into 4 quadrants with a hidden platform (diameter of 10 cm), placed in one quadrant 1 cm below the water surface. For navigation test, at the start of every trial, mice were individually positioned in the pool facing the wall. Start location differed for every trial. Each mouse was permitted 60 s for swimming to allow the location of the hidden platform. If the platform was found by a mouse, it was permitted to stay on it for 10 s. However, if the platform was not found within the 60 s, mice were guided onto the platform and permitted to rest there for 30 s. Latency time for finding the hidden platform was recorded. A probe test was then done 1 day after the last orientation navigation trial. The platform was detached and the mice permitted 60 s of swimming freely. Time spent in target quadrant and the counts of times each mouse crossed the place where hidden platforms had been placed were recorded. Behavioral tests were documented using video tracking software.

**Open-field test (OFT)**

The test was carried out in a square arena (90 cm × 90 cm × 40 cm). The mouse was placed into the center of the open field and allowed to move freely over a 5-min period. The apparatus was cleaned with 75% ethanol prior to each test session to eliminate odors. Behavioral tests were documented using video tracking software.

**Elevated plus maze (****EPM) test**

EPM apparatus was composed of polypropylene and was placed 50 cm above the floor. The apparatus was made of a cross-shaped platform comprising 2 opposing open arms (OAs, 50×10 cm) perpendicular to 2 opposing closed arms (CAs, 50×10 cm) with a small central platform (CP, 10×10 cm) between the arms. The walls of arms were 40 cm in height. Initially, every mouse was positioned at the maze’s center, facing an open arm, and was permitted 5 min of exploring the maze. Behavioral tests were documented using video tracking software.

**Sucrose preference test** **(SPT)**

Before the SPT, mice were separately housed and habituated to 48 h of forced consumption of a 1% sucrose solution from two bottles, one placed on each side of the cage. After mice adaptation, water deprivation was done for 14 h, after which they were presented with two pre-weighed bottles, one with water and the other with 1% sucrose solution, for 1 h. To avoid spatial bias, sides on which each bottle was placed was randomized. After the test, bottles were again weighed and weight differences taken as the intake from every bottle. Sucrose preferences were calculated as: sucrose intake/ (sucrose intake + water intake) × 100%.

**Forced swimming test (FST)**

Mice were independently positioned in a plastic cylinder (height: 45 cm, diameter: 25 cm) with about 35 cm of water (24 ± 1 °C). After 15 min, mice were removed from water, dried using towels, and placed in their cages. The next day, mice were again placed in the same experimental environments and subjected to 5 min of an FST. Recording of the test was done using a camera placed above the cylinder. To eliminate odors, water was renewed after each test. The observer was blinded to experimental conditions. Mice were said to be immobile when they stopped struggling in the water and floated in upright positions or only made small movements to maintain its head above water.

**Statistical analysis**

Results from the experiment were expressed as means ± SD and analyzed using SPSS software (SPSS 19.0). Differences between groups were determined by a one-way ANOVA test, followed by Tukey's test for post hoc comparisons. The prior level of significance was established at p < 0.05.
